# Nodular Cutaneous Lesions in Immune-Compromised Hosts as a Clue for the Diagnosis of Disseminated Nocardiosis: From Bedside to Microbiological Identification

**DOI:** 10.3390/pathogens12010068

**Published:** 2022-12-31

**Authors:** Ilaria De Benedetto, Antonio Curtoni, Tommaso Lupia, Simone Mornese Pinna, Silvia Scabini, Guido Ricciardelli, Marco Iannaccone, Luigi Biancone, Massimo Boffini, Mauro Mangiapia, Rossana Cavallo, Francesco Giuseppe De Rosa, Silvia Corcione

**Affiliations:** 1 Department of Medical Sciences, Infectious Diseases, University of Turin, 10126 Turin, Italy; 2 Microbiology Laboratory, “Città della Salute e della Scienza”, Hospital of Turin, 10126 Turin, Italy; 3 Infectious Diseases Unit, Cardinal Massaia Hospital, 14100 Asti, Italy; 4 Division of Nephrology Dialysis and Transplantation, Department of Medical Sciences, “Città Della Salute e Della Scienza Hospital”, University of Turin, 10126 Turin, Italy; 5 Department of Cardiac Surgery, “Città Della Salute e Della Scienza Hospital”, University of Turin, 10126 Turin, Italy; 6 Division of Pneumonology, “Città della Salute e della Scienza Hospital”, University of Turin, 10126 Turin, Italy; 7 School of Medicine, Tufts University, Boston, MA 02153, USA

**Keywords:** *Nocardia*, SOT, cutaneous, disseminated, MALDI-TOF

## Abstract

Background. *Nocardia* is a group of ubiquitous bacteria known to cause opportunistic infections in immunocompromised hosts, including those affected by malignancies and solid-organ or hematopoietic stem cell transplants. Pulmonary involvement, occurring in two-thirds of cases, is the most frequent presentation. Diagnosis might be challenging both because of microbiological technical issues, but also because of the variability of organ involvement and mimicry. Methods. We describe four cases of disseminated nocardiosis caused by *N. farcinica* observed between September 2021 and November 2021 in immune-compromised hosts presenting with nodular cutaneous lesions that had raised a high degree of clinical suspect and led to microbiological identification through MALDI-TOF MS. Results. Cutaneous involvement is typically reported in immunocompetent hosts with primary cutaneous nocardiosis with multiple forms of manifestation; nonetheless, disseminated nocardiosis rarely involves the skin and subcutaneous tissues, and this occurs as a result of metastatic spread. Our cases were disseminated nocardiosis in which the metastatic cutaneous involvement, even if rare, provided a clue for the diagnosis. Conclusions. The pathomorphosis of disseminated nocardiosis may have changed in the current years with more rapid spread due to advanced immunosuppression. For this reason, after clinical suspicion, the prompt start of an active targeted therapy based on rapid microbiological identification might potentially open the way to hopeful results, even in the most immune-compromised patients.

## 1. Background

*Nocardia* is a group of ubiquitous bacteria known to cause opportunistic infections in immunocompromised hosts [1]. That formerly known as the *Nocardia steroids* complex is responsible of the majority of infections [2] and was separated into *Nocardia abscessus*, the *Nocardia brevicatena-paucivorans* complex, the *Nocardia nova* complex (including *Nocardia nova*, *Nocardia veterana*, *Nocardia africana* and *Nocardia kruczakiae*), the *Nocardia transvalensis* complex, *Nocardia farcinica*, *Nocardia asteroids*, and *Nocardia cyriacigeorgica* [3,4].

*Nocardia* is able to grow on nonselective media under aerobic conditions [5] and on selective media such as Thayer-Martin, charcoal buffered yeast extract, Sabouraud glucose agar, and Lowenstein-Jensen media, minimizing the overgrowth of contaminating microorganisms. Microscopically they appear as variable Gram-positive, catalase-positive thin, branching, filamentous rods [3,5]. Modified Ziehl-Neelsen stain in most cases is negative, whereas on modified Kinyoun, stain *Nocardia* is variably acid-fast [5]. Colonies are typically dry, chalky white with aerial hyphae (0.5–1.2 μm in diameter), but they can also appear as smooth, turning to orange with age, such as with *N. farcinica*, or yellowish, tan, brown in relation to the production of soluble brown or yellow pigments. Since the growth is usually clear from 2–7 days up to several weeks, it is essential, in cases of clinical suspicion, to inform the laboratory so that correct media and incubation protocols can be applied [5,6].

*Nocardia* infection typically occurs in cell-mediated immune-depressed patients, such as those with malignancies, human immunodeficiency virus infection, solid-organ or hematopoietic stem cell transplants and treated with long-term steroids or cell-mediated immunomodulators [7,8]. In hematopoietic stem cell transplants, the timing of the possible development of nocardiosis ranges from 2–3 months up to 2 years [9,10], whereas in solid organ transplanted (SOT) patients, onset occurs in two-thirds of cases within one year from transplant. In solid organ transplanted patients, the prevalence of nocardiosis is 0.7–3% [11,12,13,14,15], and the highest frequency is reported for lung transplants, followed by that with heart, intestinal, kidney and liver transplants. Among those, two-thirds of patients have isolated pulmonary disease and 20% have disseminated disease, and the most common isolates are *N. nova* in half of cases, followed by *N. farcinica* in 30% [6,15]. The diagnosis of nocardiosis might be challenging not only due to microbiological technical issues, but also because of the variability of organ involvement and the differentials with mimicries, such as tuberculosis, aspergillosis, or actinomycosis. 

## 2. Methods

We describe four cases of disseminated nocardiosis caused by *N. farcinica* observed between September 2021 and November 2021 in immune-compromised hosts in a 1200-bed academic hospital with primary and secondary referral (City of Health and Sciences, Molinette Hospital, Turin, Italy) presenting with nodular cutaneous lesions that had raised a high degree of clinical suspect and led to microbiological identification (Table 1). In our cases, clinical samples were microscopically screened via Gram-staining. *Nocardia* isolation was performed on Columbia agar with 5% sheep blood with an incubation protocol of 7–10 days at 37 °C in aerobic conditions. Suggestive colonies (macroscopic features) were identified via Matrix-Assisted Laser Desorption/Ionization Time-Of-Flight Mass Spectrometry (MALDI-TOF MS) on a Bruker Microflex LT with the MBT 8468 MSP 2019 Library (Bruker Daltonics, Bremen, Germany). Antimicrobial susceptibility to amoxicillin/clavulanate, imipenem, ciprofloxacin, linezolid, amikacin, tobramycin and TMP/SMX was determined through an ETEST (BioMérieux, Paris, France) on Mueller-Hinton Fastidious Agar after 48–72 h at 37 °C. Results were interpreted according to CLSI criteria [16]. Written informed consent was obtained from patients for publication. 

## 3. Results

**Figure 1:**
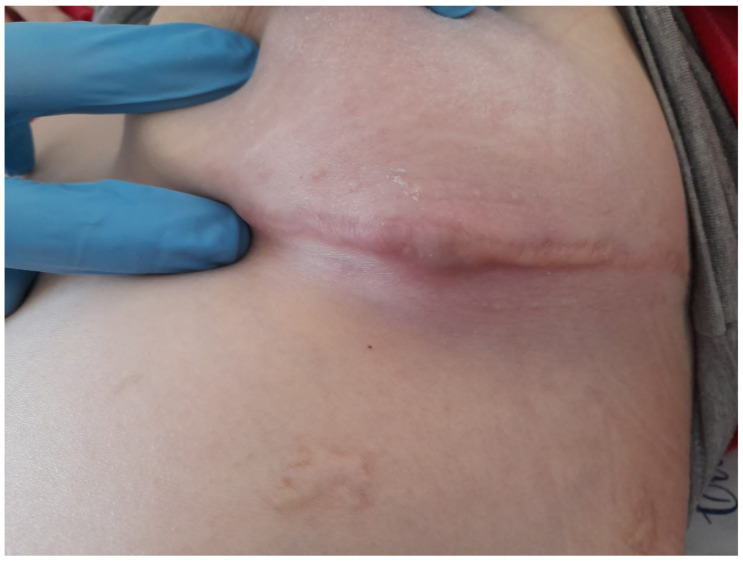
Left sub-mammary painless cutaneous and subcutaneous abscess with erythematosus infiltration of the surgical scar in a patient with cystic fibrosis and bilateral lung transplant.

**Case 1.** A 21-year-old female with cystic fibrosis and bilateral lung transplant 2 months prior presented with fever, cough, desaturation and a left sub-mammary painless cutaneous and subcutaneous abscess with erythematosus infiltration of the previous surgical scar (Figure 1). Tacrolimus, mycofenolate mofetil and low-dose steroids were the current immunosuppressive regimen together with acyclovir and trimethoprim/sulfamethoxazole (TMP/SMX) prophylaxis. A chest CT scan showed a consolidation in the right inferior lobe with necrotic–colliquative areas and nodular lesions, colliquative mediastinic lymphadenopathies, an ipodense, polylobate with an iperdense wall subcutaneous right sub-mammary lesion and hepatic and renal lesions. *N. farcinica* was isolated both from the drainage of cutaneous abscesses and bronchoalveolar lavage. Antibiotic treatment with intravenous TMP/SMX, amikacin and ertapenem was started, which was then switched to linezolid, amikacin and meropenem due to an antimicrobial susceptibility test showing resistance to TMP/SMX. Because of the rapid deterioration of the patient’s clinical condition with hemodynamic instability and persistent fever, a rescue therapy with tigecycline adjunction was tried with a beneficial effect. A diagnosis of post-transplant lymphoproliferative disorder was made, and rituximab was started with the remission of fever. The patient has now completed two out of nine months of antibiotic treatment for disseminated nocardiosis (Table 1).

**Figure 2:**
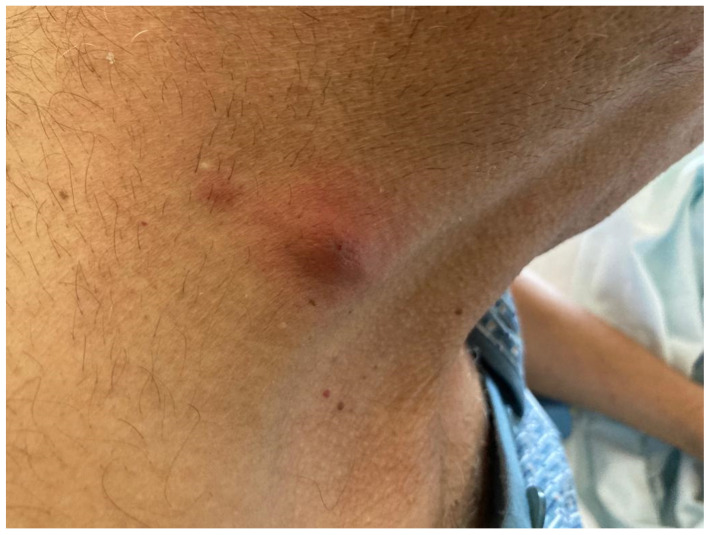
Right axillar nodular, thick and slightly painful cutaneous and subcutaneous lesion with a surrounding erythematosus halo in a heart-transplanted patient with disseminated nocardiosis.

**Figure 3:**
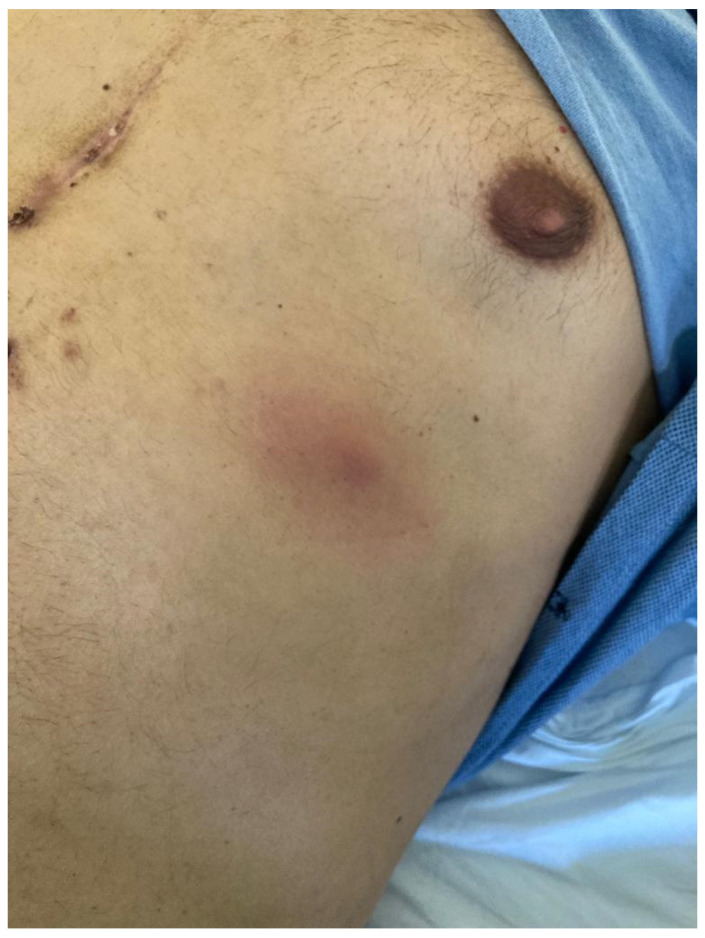
Left thoracic, thick and slightly painful cutaneous and subcutaneous lesion with a surrounding erythematosus halo in a heart-transplanted patient with disseminated nocardiosis.

**Case 2.** A 54-year-old male with a heart transplant 1.5 months prior presented with fever and a follow-up chest CT scan showing four small nodular lesions. During physical examination, right axillar, left thoracic and left medial lower limb nodular, thick and slightly painful cutaneous and subcutaneous lesions with a surrounding erythematosus halo were observed (Figure 2 and Figure 3). Because of the clinical suspicion of disseminated nocardiosis or aspergillosis, bronchoalveolar lavage and a cutaneous biopsy were performed, and an empiric treatment with liposomal B amphotericin and intravenous TMP/SMX was started. *N. farcinica* was isolated from bronchoalveolar lavage, whereas the galactomannan antigen tested negative. Liposomal B amphotericin was stopped, and according to the antibiogram susceptibility test, a combination of target therapy with amikacin and intravenous TMP/SMX was administered for two weeks during hospitalization. At discharge, following an oral combination therapy with linezolid and TMP/SMX that continued for two weeks, the simplification to oral TMP/SMX was prescribed. The patient has now completed two out of nine months of antibiotic treatment for disseminated nocardiosis (Table 1).

**Case 3.** A 74-year-old female with a kidney transplant 7 months prior presented with fever and cough. Since community-associated pneumonia was suspected, antibiotic treatment with ceftriaxone and azithromycin was started. Because of the poor clinical response, after two days, the treatment was switched to ceftobiprole and azithromycin for 6 days. A bronchoalveolar lavage culture tested positive for *N. farcinica.* A chest CT scan was performed and showed consolidation in the left inferior lobe with a 28 mm colliquative solid lesion and numerous small bilateral nodulations. Antibiotic treatment with intravenous TMP/SMX was started, but because of deteriorating renal function after one week and a cerebral CT scan showing an incidental 3 mm parietal nodular lesion, a switch to linezolid was proposed. The patient has now completed one out of nine months of antibiotic treatment for disseminated nocardiosis (Table 1).

Case 4. A 66-year-old male with an inoperable biliary duct carcinoma treated with palliative chemotherapy with gemcitabine and cisplatin presented with fever, cough and desaturation and a nodular cutaneous ecchymosis on the left knee. A bronchoalveolar lavage culture tested positive for *N. farcinica*, after other bacterial and fungal causes were excluded. An antimicrobial susceptibility test showed resistance to TMP/SMX and amikacin, and antibiotic treatment with imipenem/cilastatin and linezolid was started. The patient has now completed two out of nine months of antibiotic treatment for disseminated nocardiosis (Table 1).

## 4. Conclusions

We describe four cases of disseminated nocardiosis with initial nodular cutaneous lesions in immune-compromised hosts together with pulmonary involvement. Cutaneous involvement is typically reported in immunocompetent hosts with primary cutaneous nocardiosis with multiple forms of manifestation, such as a superficial cellulitis occurring via direct inoculation, abscess, lymphocutaneous satellite infection similar to sporotrichosis or mycetoma with the formation of a sinus tract and a more destructive disease. Nonetheless, disseminated nocardiosis rarely involves the skin and subcutaneous tissues, and this occurs as a result of a metastatic spread [17,18,19]. Our cases were disseminated nocardiosis in which the metastatic cutaneous involvement, even if rare, provided a clue for the diagnosis. In fact, pulmonary involvement was the most frequent representation in a recent work by Galar et al. [20] over 24 years of retrospective observation, and in particular, the incidence of disseminate nocardiosis is described as stable with a decrease in HIV and SOT patients and an increase in elderly patients with chronic respiratory conditions and corticosteroid treatment. Similarly, in our experience, even though the clinical suggestion came from the dermatological findings, at the same time, pulmonary involvement was present in all cases. Other studies have reported an increasing incidence of nocardiosis even though these studies lack information about the last ten years and in particular about the incidence after the introduction of new technologies [21,22,23], such as the implementation of MALDI-TOF MS, which might have improved the diagnostic yield with an increasing number of identified nocardiosis cases and in particular a more rapid time of response from the laboratory to the clinicians, helping to attribute the etiology of unusual clinical findings or mimicries of different diseases to *Nocardia* isolates. In fact, traditionally, *Nocardia* species identification was based on biochemical tests; the long response time, the lack of commercially available kits and the difficulties in their application in Clinical Microbiology Laboratories contributed to their progressive disuse in favor of new technologies [24,25]. MALDI-TOF has revolutionized the routine identification of microorganisms, including *Nocardia* [21,23]. Recently, this tool has been shown to provide reliable *Nocardia* species identification within a few minutes rather than several days, starting from isolated colonies [26,27,28,29].

In our cases, prompt identification was obtained in 48 h, with the start of a targeted therapy following antimicrobial susceptibility tests for nocardiosis within 72 h, solving the well-known issue of long incubation times and interference by fast-growing microorganisms [27]. This is the reason why the diagnosis of nocardiosis depends on a close collaboration among physicians and clinical microbiologists. Moreover, *Nocardia* microbiological identification at the species-level is mandatory to interpret clinically significant isolates and to administer correct antimicrobial therapy since *Nocardia* drug resistance patterns are largely species-related [28,29,30,31]. For this purpose, because of the shortcomings of the commercial MALDI-TOF MS database for the identification of *Nocardia* species, further identification based on the complete sequence of the gold standard 16S rRNA is recommended to confirm species assignment [32]. In fact, *N. farcinica* together with the *N. transvalensis* complex, *N. otitidiscaviarum* and the *N. nova* complex *N. farcinica* is one of the multidrug-resistant (MDR) species reported in the literature [29,30,31]. Not all studies, however, agree on resistance percentages. Drug susceptibility differences reported in the literature could be ascribed to technical difficulties, such as standard inoculum preparation, laboratory testing methodology, result reading and interpretation criteria [33]. Local epidemiological variation is another important factor to keep in mind for data comparisons; for this reason, even if identification provides important information to switch from empirical to a targeted antimicrobial therapy, antimicrobial susceptibility testing should always be performed [34].

The *N. farcinica* drug pattern is usually characterized by resistance to ceftriaxone, tobramycin and clarithromycin and by susceptibility to TMP/SMX, amoxicillin/clavulanate, ciprofloxacin, linezolid and amikacin [27]. In our casuistry, *N. farcinica* antimicrobial susceptibility testing was similar to the expected one. Linezolid was the only effective drug for all bacterial isolates, followed by amoxicillin/clavulanate, imipenem and amikacin, which were active in 3/4 cases. The only two TMP/SMX-resistant strains were MDR, according to the criteria of Magiorakos et al. [34]. In recent work by Goodlet et al. [35], previous TMP/SMX prophylaxis in lung transplant patients has been found to be an independent protective factor for nocardiosis; nonetheless, one of our patients who received TMP/SMX prophylaxis developed an infection sustained by a resistant strain.

Even though available follow-up information is limited in our cases and long-term outcomes are not known, we assisted in the rapid improvement in patients’ conditions after the start of the targeted treatment. These data can be explained by the relatively short length of disease since all patients presented with the acute onset of clinical signs; nonetheless, in all of our solid organ-transplanted patients, disseminated nocardiosis occurred in the first year post-transplantation in the period of maximal immune depression as previously shown [17,36,37,38]. Even though disseminated nocardiosis is a well-known infective complication in immune-depressed and transplant patients, the pathomorphosis of disseminated nocardiosis may have changed in the current years with more rapid spread due to advanced immunosuppression. In our opinion, it is important to keep high clinical suspicion in cases of typical cutaneous lesions because the prompt start of an active targeted therapy based on rapid microbiological identification might potentially open the way to hopeful results even for the most immune-compromised patients.

## Figures and Tables

**Table 1:** Description of four immune-compromised patients with disseminated nocardiosis.

Patients	Sex	Isolates	Antimicrobial Susceptibility	Predisposing Condition	Nocardiosis Localization	Treatment	Outcome
21 years-old	F	*Nocardia farcinica*	amox./clav. R imipenem R ciprofloxacin R linezolid S amikacin S tobramycin R TMP/SMX R	Lung transplant for cystic fibrosis	Cutaneous, pulmonary, lymphonodal, hepatic and renal	**Empirical:** TMP/SMX, amikacin and ertapenem **Targeted:** linezolid, amikacin and meropenem **Rescue:** linezolid, amikacin, meropenem and tygecicline	On treatment (two months completed)
54 years-old	M	*Nocardia farcinica*	amox./clav. R imipenem S ciprofloxacin R linezolid S amikacin S tobramycin R TMP/SMX S	Heart transplant	Cutaneous and pulmonary	**Empirical:** TMP/SMX **Targeted:** amikacin and TMP/SMX, then linezolid and TMP/SMX, followed by TMP/SMX	On treatment (two months completed)
74 years-old	F	*Nocardia farcinica*	amox./clav. R imipenem S ciprofloxacin S linezolid S amikacin S tobramycin R TMP/SMX S	Kidney transplant	Cutaneous, pulmonary and cerebral	**Empirical:** none **Targeted:** TMP/SMX, then linezolid	On treatment (one month completed)
66 years-old	M	*Nocardia farcinica*	amox./clav. R imipenem R ciprofloxacin R linezolid S amikacin S tobramycin R TMP/SMX R	Biliary duct carcinoma with palliative chemotherapy	Cutaneous and pulmonary	**Empirical:** none **Targeted:** imipenem/cilastatin and linezolid	On treatment (two months completed)

## Data Availability

Not applicable.

## References

[1] Conville P.S., Brown-Elliott B.A., Smith T., Zelazny A.M. (2018). The Complexities of *Nocardia* Taxonomy and Identification. J. Clin. Microbiol..

[2] Wallace R.J., Steele L.C., Sumter G., Smith J.M. (1988). Antimicrobial susceptibility patterns of *Nocardia asteroides*. Antimicrob. Agents Chemother..

[3] Brown-Elliott B.A., Brown J.M., Conville P.S., Wallace R.J. (2006). Clinical and laboratory features of the *Nocardia* spp. based on current molecular taxonomy. Clin. Microbiol. Rev..

[4] Schlaberg R., Huard R.C., Della-Latta P. (2008). *Nocardia cyriacigeorgica*, an emerging pathogen in the United States. J. Clin. Microbiol..

[5] NHS Institute (2016). UK Standards for Microbiology Investigations. Identification of Aerobic Actinomycetes. https://www.gov.uk/government/publications/smi-id-10-identification-of-aerobic-actinomycetes.

[6] Saullo J.L., Miller R.A. (2020). Update on Nocardia infections in solid-organ transplantation. Curr. Opin. Organ Transplant..

[7] Young L.S., Rubin R.H., Rubin R.H., Young L.S. (2002). Mycobacterial and nocardial diseases in the compromised host. A Clinical Approach to Infection in the Compromised Host.

[8] Long P.F. (1994). A retrospective study of *Nocardia* infections associated with the acquired immune deficiency syndrome (AIDS). Infection.

[9] Choucino C., Goodman S.A., Greer J.P., Stein R.S., Wolff S.N., Dummer J.S. (1996). Nocardial Infections in Bone Marrow Transplant Recipients. Clin. Infect. Dis..

[10] Van Burik J., Hackman R.C., Nadeem S.Q., Hiemenz J.W., White M.H., Flowers M.E.D., Bowden R.A. (1997). Nocardiosis After Bone Marrow Transplantation: A Retrospective Study. Clin. Infect. Dis..

[11] (2004). Nocardia infections. Am. J. Transpl..

[12] Forbes G., Harvey F., Philpott-Howard J., O’Grady J., Jensen R., Sahathevan M., Casewell M., Williams R. (1990). Nocardiosis in liver transplantation: Variation in presentation, diagnosis and therapy. J. Infect..

[13] Simpson G.L., Stinson E.B., Egger M.J., Remington J.S. (1981). Nocardial infections in the immunocompromised host: A detailed study in a defined population. Rev. Infect. Dis..

[14] Wilson J.P., Turner H.R., Kirchner K.A., Chapman S.W. (1989). Nocardial infections in renal transplant recipients. Medicine.

[15] Peleg A.Y., Husain S., Qureshi Z.A., Silveira F.P., Sarumi M., Shutt K.A., Kwak E.J., Paterson D.L. (2007). Risk Factors, Clinical Characteristics, and Outcome of Nocardia Infection in Organ Transplant Recipients: A Matched Case-Control Study. Clin. Infect. Dis..

[16] CLSI (2018). Susceptibility Testing of Mycobacteria, Nocardia spp., and Other Aerobic Actinomycetes.

[17] Wilson J.W. (2012). Nocardiosis: Updates and Clinical Overview. Mayo Clin. Proc..

[18] Outhread A.C., Watts M.R., Chen S.C., Sorrell T.C. (2011). Nocardia Infections of the Face and Neck. Curr. Infect. Dis. Rep..

[19] Wang H.L., Seo Y.H., LaSala P.R., Tarrand J.J., Han X.Y. (2014). Nocardiosis in 132 patients with cancer: Microbiological and clinical analyses. Am. J. Clin. Pathol..

[20] Galar A., Martín-Rabadán P., Marín M., Cercenado E., Sánchez-Carrillo C., Valerio M., Bouza E., Muñoz P. (2020). Revisiting nocardiosis at a tertiary care institution: Any change in recent years?. Int. J. Infect. Dis..

[21] Croxatto A., Prod’hom G., Greub G. (2012). Applications of MALDI-TOF Mass Spectrometry in Clinical Diagnostic Microbiology. FEMS Microbiol. Rev..

[22] Verroken A., Janssens M., Berhin C., Bogaerts P., Huang T.D., Wauters G., Glupczynski Y. (2010). Evaluation of Matrix-Assisted Laser Desorption Ionization-Time of Flight Mass Spectrometry for Identification of *Nocardia* Species. J. Clin. Microbiol..

[23] Ambrosioni J., Lew D., Garbino J. (2010). Nocardiosis: Updated clinical review and experience at a tertiary center. Infection.

[24] Boiron P., Provost F., Chevrier G., Dupont B. (1992). Review of nocardial infections in France 1987 to 1990. Eur. J. Clin. Microbiol. Infect. Dis..

[25] Tremblay J., Thibert L., Alarie I., Valiquette L., Pepin J. (2011). Nocardiosis in Quebec, Canada, 1988–2008. Clin. Microbiol. Infect..

[26] Lafont E., Conan P.L., Rodriguez-Nava V., Lebeaux D. (2020). Invasive Nocardiosis: Disease Presentation, Diagnosis and Treatment—Old Questions, New Answers?. Infect. Drug Resist..

[27] Wei M., Wang P., Qu J., Li R., Liu Y., Gu L., Yang C. (2017). Identification and antimicrobial susceptibility of clinical Nocardia species in a tertiary hospital in China. J. Glob. Antimicrob. Resist..

[28] Valdezate S., Garrido N., Carrasco G., Medina-Pascual M.J., Villalón P., Navarro A.M., Saéz-Nieto J.A. (2016). Epidemiology and susceptibility to antimicrobial agents of the main *Nocardia* species in Spain. J. Antimicrob. Chemother..

[29] Lebeaux D., Bergeron E., Berthet J., Djadi-Prat J., Mouniée D., Boiron P., Lortholary O., Rodriguez-Nava V. (2018). Antibiotic susceptibility testing and species identification of Nocardia isolates: A retrospective analysis of data from a French expert laboratory, 2010–2015. Clin. Microbiol. Infect..

[30] Larruskain J., Idigoras P., Marimón J.M., Pérez-Trallero E. (2011). Susceptibility of 186 Nocardia sp. Isolates to 20 Antimicrobial Agents. Antimicrob. Agents Chemother..

[31] Kuo S.-F., Chen F.-J., Lan I.-C., Chien C.-C., Lee C.-H. (2022). Epidemiology of *Nocardia* Species at a Tertiary Hospital in Southern Taiwan, 2012 to 2020: MLSA Phylogeny and Antimicrobial Susceptibility. Antibiotics.

[32] Shahraki A.H., Heidarieh P., Bostanabad S.Z., Hashemzadeh M., Feizabadi M.M., Schraufnagel D., Mirsaeidi M. (2015). Genetic diversity and antimicrobial susceptibility of Nocardia species among patients with nocardiosis. Sci. Rep..

[33] Tan Y.E., Chen S.C., Halliday C.L. (2019). Antimicrobial susceptibility profiles and species distribution of medically relevant Nocardia species: Results from a large tertiary laboratory in Australia. J. Glob. Antimicrob. Resist..

[34] Magiorakos A.-P., Srinivasan A., Carey R.B., Carmeli Y., Falagas M.E., Giske C.G., Harbarth S., Hindler J.F., Kahlmeter G., Olsson-Liljequist B. (2012). Multidrug-resistant, extensively drug-resistant and pandrug-resistant bacteria: An international expert proposal for interim standard definitions for acquired resistance. Clin. Microbiol. Infect..

[35] Goodlet K.J., Tokman S., Nasar A., Cherrier L., Walia R., Nailor M.D. (2020). Nocardia prophylaxis, treatment, and outcomes of infection in lung transplant recipients: A matched case-control study. Transpl. Infect. Dis..

[36] Minero M.V., Marin M., Cercenado E., Rabadan P.M., Bouza E., Munoz P. (2009). Nocardiosis at the turn of the century. Medicine.

[37] Tripodi M.-F., Durante-Mangoni E., Fortunato R., Cuccurullo S., Mikami Y., Farina C., Utili R. (2010). In vitro activity of multiple antibiotic combinations against Nocardia: Relationship with a short-term treatment strategy in heart transplant recipients with pulmonary nocardiosis. Transpl. Infect. Dis..

[38] Fishman J.A. (2007). Infection in solid-organ transplant recipients. N. Engl. J. Med..

